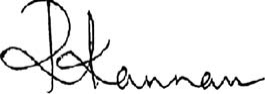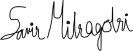# Engineering clinical translation‐Introduction to Special Issue Dedicated to 2017 Bioengineering and Translational Medicine Conference

**DOI:** 10.1002/btm2.10099

**Published:** 2018-07-27

**Authors:** Rangaramanujam M. Kannan, Samir Mitragotri

**Affiliations:** ^1^ Johns Hopkins University, School of Medicine; ^2^ Harvard University

A timely convergence of many factors, including gene‐editing technologies, machine learning, nanomedicine, and artificial intelligence, is making it tremendously exciting to be an engineer at the interface of medicine. This is further fueled by the recognition that we need bold strategies to tackle health care challenges across the world including ways to prevent autism and Alzheimer's, making medicines economical, and speeding up the process of translation. Bringing perspectives from across the spectrum of stakeholders is critical to speeding up translation. Engineers are well positioned be an enabling community in this regard. Recognizing this opportunity, the American Institute of Chemical Engineers (AIChE) and Society of Biological Engineers (SBE) launched this journal and the conference series starting in 2016. The second conference in this series was held in Minneapolis on the heels of the annual AIChE meeting from October 28–29 (Co‐chairs: Ali Khademhosseini and Efie Kokkoli), featuring exciting talks and posters covering focus areas such as stem cells/regenerative medicine, immunoengineering, regulatory aspects, biopharmaceuticals and gene/drug delivery. These are being highlighted in this issue edited by Rangaramanujam Kannan (Johns Hopkins Medicine) and Samir Mitragotri (Harvard).

2017 marked the year when multiple gene therapy products were successful. *William Kaemmerer* reviews the landscape and outlines the challenges for the field to make a bigger impact, including the need for manufacturing capacity improvements for viral vectors. *Vargasan and Anselmo* review the opportunities for the next generation microbe‐based therapies that are undergoing clinical translation, including genetically‐engineered microbes. *Sharma, Kannan and coworkers* describe an example of defining the nanoproduct and enabling the translation from lab scale to manufacturing of the first dendrimer‐drug conjugate for the systemic treatment of neuroinflammation in CNS disorders, undergoing human trials. *Jarvis, Mitragotri and coworkers* underscore the importance of understanding the physiological stability of ligand‐modified nanoparticles. Using microfluidic devices, they illustrate how endothelial cell contact, conjugation chemistry, and vascular flow parameters and conjugation can modify surface ligands on nanoparticle surfaces.

*Knapp, Whitehead and coworkers* describe the approach of delivering RNAi cocktails using lipid nanoparticles for the eventual treatment of Mantle cell lymphoma, with significant potential in aggressive tumors. *Monroe, Flexner and Cui* define the next challenges associated with antiretroviral therapy, not only preventing viral replication and progression to AIDS, but killing the latent virus. The potential of nanoparticle‐based systems in improving tissue penetration and targeting of the latent virus, enabling reduce drug doses at lower dosing frequency are explored. *Hou, Chen and coworkers* describe what could the new wave of T‐cell immunotherapy approaches where a new class of TGF_β_—promoting chimeric antigen receptor T‐cells can play a key role in adoptive T‐cell therapy. They underscore the importance of understanding the interaction between these cells and the effector and regulatory T‐cells. *Howsman, Hahn and coworkers* describe a multivariate technique that can enable early diagnosis of autism spectrum disorders and provide biochemical understanding of the disease.

In many ways, the articles in the first of the two conference special issues, and the BTM conference highlighted the rich variety and depth of perspectives bioengineers can bring to important health issues, through their collaborations with physicians, regulators and investors. We may be at the tipping point where such perspectives can accelerate translation of discoveries, human impacting health meaningfully.